# A holistic comparative analysis of diagnostic tests for urothelial carcinoma: a study of Cxbladder Detect, UroVysion® FISH, NMP22® and cytology based on imputation of multiple datasets

**DOI:** 10.1186/s12874-015-0036-8

**Published:** 2015-05-12

**Authors:** Vivienne Breen, Nikola Kasabov, Ashish M. Kamat, Elsie Jacobson, James M. Suttie, Paul J. O’Sullivan, Laimonis Kavalieris, David G. Darling

**Affiliations:** Auckland University of Technology, Auckland, New Zealand; M. D. Anderson Cancer Center, University of Texas, Houston TX, USA; Pacific Edge Limited, Dunedin, New Zealand

**Keywords:** Cancer diagnostic tests ranking, Diagnostic test accuracy, Multiple data integration, Data imputation, Urothelial carcinoma, Urine cytology, NMP22, FISH, Cxbladder detect

## Abstract

**Background:**

Comparing the relative utility of diagnostic tests is challenging when available datasets are small, partial or incomplete. The analytical leverage associated with a large sample size can be gained by integrating several small datasets to enable effective and accurate across-dataset comparisons. Accordingly, we propose a methodology for a holistic comparative analysis and ranking of cancer diagnostic tests through dataset integration and imputation of missing values, using urothelial carcinoma (UC) as a case study.

**Methods:**

Five datasets comprising samples from 939 subjects, including 89 with UC, where up to four diagnostic tests (cytology, NMP22®, UroVysion® Fluorescence *In-Situ* Hybridization (FISH) and Cxbladder Detect) were integrated into a single dataset containing all measured records and missing values. The tests were firstly ranked using three criteria: sensitivity, specificity and a standard variable (feature) ranking method popularly known as signal-to-noise ratio (SNR) index derived from the mean values for all subjects clinically known to have UC versus healthy subjects. Secondly, step-wise unsupervised and supervised imputation (the latter accounting for the ‘clinical truth’ as determined by cystoscopy) was performed using personalized modelling, *k*-nearest-neighbour methods, multiple logistic regression and multilayer perceptron neural networks. All imputation models were cross-validated by comparing their post-imputation predictive accuracy for UC with their pre-imputation accuracy. Finally, the post-imputation tests were re-ranked using the same three criteria.

**Results:**

In both measured and imputed data sets, Cxbladder Detect ranked higher for sensitivity, and urine cytology a higher specificity, when compared with other UC tests. Cxbladder Detect consistently ranked higher than FISH and all other tests when SNR analyses were performed on measured, unsupervised and supervised imputed datasets. Supervised imputation resulted in a smaller cross-validation error. Cxbladder Detect was robust to imputation showing a 2 % difference in its predictive versus clinical accuracy, outperforming FISH, NMP22 and cytology.

**Conclusion:**

All data analysed, pre- and post-imputation showed that Cxbladder Detect had higher SNR and outperformed all other comparator tests, including FISH. The methodology developed and validated for comparative ranking of the diagnostic tests for detecting UC, may be further applied to other cancer diagnostic datasets across population groups and multiple datasets.

**Electronic supplementary material:**

The online version of this article (doi:10.1186/s12874-015-0036-8) contains supplementary material, which is available to authorized users.

## Background

Currently there are no effective information science methods for comparing and ranking diagnostic test performance across sample populations, particularly when different combinations of diagnostic tests are compared in different studies with different populations. Furthermore, comparisons are challenging when missing values are present in each sample population.

Data imputation has previously been used to successfully manage missing data in several cancer studies. This has been particularly successful where one or more common variables are present across datasets. Population-based studies, particularly those analysing records where data are incomplete, benefit from multiple imputation by permitting a fuller analysis of incomplete records [1]. For example, Nur et al. [1] used imputation techniques to refine mortality estimates by including stage, morphology and grade data for colorectal cancer patients from an additional 45 % of the patient cohort where data were incomplete. Other studies have also indicated that imputation can be used to manage missing clinical data, for example, tumor stage, from patients with colorectal, lung and breast cancers and melanoma [2–5].

In addition, genotype imputation is already used in the analysis of genome-wide association scans [6]. This technique involves imputing the genotypes of unsequenced parts of the genome based on data from more fully sequenced reference individuals and is particularly useful if data are combined from studies that used different sequencing panels across different populations. A variety of other examples of imputation in biological and clinical data and different modelling approaches to this data can be found [7].

Various statistical and machine learning methods for data imputation have been proposed and applied so far. Rubin [8, 9] and Little and Rubin [10] provide an overview of statistical methods for multiple imputation and analysis of data with missing values. Su et al. [11] provides Bayesian methods, multiple imputation and model diagnostics. The Markov chain MonteCarlo (MCMC) approach to Bayesian modelling estimates the conditional distribution of model parameters given the observed data and the prior parameter distribution (the a-posteriori distribution) [12]. This approach may be interpreted as a multiple imputation procedure where many sets of missing observations are generated from their a-posteriori distribution. However, the imputed data sets do not play a direct role in the estimation of parameters as in the classical approach developed by Little and Rubin [10].

The majority of patients with UC present with urological symptoms, such as macroscopic hematuria (visible blood in the urine), microscopic hematuria (≥3 red blood cells per high-powered field) or irritative voiding in the absence of a benign cause. The current standard of care for diagnosing these patients is cystoscopy and pathological examination of biopsies [13].

A number of non-invasive urine tests are now available that can be used as an adjunct to, or in low-risk cases, a replacement for, investigative cystoscopy. In urine cytology, cells present in voided urine or bladder wash samples are examined and described as being positive or negative for the presence of malignant cells, atypical or having suspicious cells present [14]. NMP22® is a nuclear mitotic protein involved in chromatin segregation that is used to diagnose patients with UC in two urinary assays, a reference laboratory enzyme immunoassay (ELISA) and a cassette point-of-care test (NMP22 BladderChek®). A cut-off level assessed by the NMP22 test kit has been validated to distinguish positive from negative results [15]. UroVysion® Fluorescence *In-Situ* Hybridization (FISH) is a urine-based test that detects aneuploidy of chromosomes 3, 7 and 17, and loss of both 9p21 loci in malignant urothelial cells from voided urine samples [16, 17]. Changes in these chromosomes correlate with the transition from normal urothelium to carcinoma, tumor progression and pathological stage and grade. FISH is not generally used to diagnose primary UC, but is applied as a reflex test for atypical cytology in a monitoring for recurrence setting. Cxbladder Detect is a gene expression test, which quantifies five mRNA biomarkers found in urine: four biomarkers (*IGFBP5, HOXA13, MDK* and *CDK1*) are associated with the growth and propagation of tumor tissue, whereas the fifth biomarker (*CXCR2*) is a marker of inflammation that is used to reduce false-positive results by identifying patients with non-malignant inflammatory conditions [18]. The relative performance of Cxbladder Detect, NMP22 Bladderchek and NMP22 ELISA have been prospectively compared with all tests offering comparable specificity [18], but no comprehensive analysis has been attempted on all currently available non-invasive urine tests because no study published to date has simultaneously assessed all tests.

In this study, we propose a methodology for comparative analysis and ranking of diagnostic tests across population groups, by integrating datasets and imputing data using datasets from sample populations. We have applied this methodology to the diagnosis of UC using urine cytology, NMP22, FISH and Cxbladder Detect because there have been few broad multi-test head-to-head comparisons between urinary tests for UC, and the varying population demographics, sample sizes and methodologies used across studies have made comparing and interpreting data difficult.

A data imputation method has considerable appeal in making a comprehensive comparison possible. More specifically, in the present study, we propose a methodology for globally ranking and comparing the accuracy of different diagnostic tests when each test has only been applied to a subgroup of patients. The novel approach presented here uses measured values from the integrated dataset to impute values for other UC tests in the same subject. As each test measures a somewhat different aspect of human pathophysiology, the comparative analysis is truly of a holistic nature.

## Methods

### Datasets

Five datasets, all owned by the authors of the manuscript, consisting of 939 patients obtained from different populations of patients who had either presented with macrohematuria at their primary diagnosis (Datasets 1–3) or for surveillance for UC recurrence (Datasets 4 and 5) were available for the study (Table 1). Some individual data points were missing in each of the datasets where not all of the tests analysed in this study were used for each of the subpopulations on all patients. Up to four diagnostic UC tests were performed in each study and for this analysis all tests were treated as having a binary outcome of being positive or negative for UC (see below). Any patient samples lacking a diagnosis based on cystoscopy as the gold standard (i.e. in the absence of a record of clinical truth), or where only one test result was available, were discarded. Patients with a diagnosis of other causes, e.g. kidney stones, were reclassified as a non-UC diagnosis alongside patients whose diagnosis was normal. All datasets were combined into a single integrated dataset containing all records, including samples with missing values.

**Table 1:** UC diagnostic test datasets used in the analysis

Dataset	Study/publication	Original dataset, n	Data analyzed (UC/non-UC)	Cytology	NMP22	FISH	Cxbladder Detect
1	Pacific Edge Limited, NZ [18]	476, Primary detection	63/411	•	•		•
2	Canterbury Urology Research Trust, Canterbury, NZ (Pacific Edge Limited, Unpublished data)	94, Primary detection	6/74	•			•
3	North Shore Hospital, Takapuna, NZ (Pacific Edge Limited, Unpublished data)	84, Primary detection	5/63	•			•
4	Kamat, USA [19]	200, Secondary monitoring	6/187	•	•	•	
5	Clinical Trials USA (Pacific Edge Limited, Unpublished data)	124, Secondary monitoring	9/115	•	•	•	•

The closed symbol (•) indicates that the test was carried out in the study. A gap indicates that the test was not carried out. Data analyzed differs from the study population as any patient samples either without diagnosis or where only one test result was available were discarded. Primary detection means the study population was composed of patients presenting with hematuria prior to UC diagnosis. Secondary monitoring means that patients were presenting after primary UC diagnosis and treatment

In Datasets 1–3 and 5, cytology and NMP22 were measured using the methods described by O’Sullivan et al. [18] and Cxbladder Detect was measured using a method based on O’Sullivan et al. [18]. The methods used to measure cytology and NMP22 in Dataset 4 were described in [19]. FISH was measured in Datasets 4 and 5 according to the manufacturer’s instructions.

Original outcomes were used for all tests and designated as either ‘positive’ or ‘negative’ for UC. For urine cytology, negative and atypical results were considered negative for UC (coded as 1 and 2, respectively), while positive and suspicious results were considered positive for UC (coded as 3 and 4, respectively) for the initial development of the integrated dataset and for the accuracy analysis (see below for details of the statistical handling of the data). For NMP22, a score of <10U was considered negative (coded as 1), while a score of ≥10U was considered positive (coded as 2) and the binary classification was used for all analyses. Positive and negative results for FISH were defined according to the manufacturer’s instructions and the binary classification was used for all analyses (coded as 2 and 1, respectively). A Cxbladder Detect result of ‘Low’ was classed as negative (coded as 1) and results of ‘Elevated’ and ‘High’ were classed as positive for the imputation and accuracy analysis (coded as 2). Two further variables that may be predictive of UC, age and gender, were also included in the initial integrated and imputation analysis.

### The proposed methodology

The proposed methodology in this study includes several well-known computational methods and procedures performed and interpreted in unique combinations:

#### Signal-to-noise ratio (SNR) ranking of variables

The discriminative power of each diagnostic test (variable) to separate samples from patients with and without UC across all samples from the integrated datasets was calculated using a feature ranking technique, popularly known as SNR (see Additional file 1 for the mathematical formula). Mean values from patients with UC were considered as ‘signal’ and mean values from patients without UC as ‘noise’. For each test, an index of separation was calculated using the difference between the mean test value of samples from patients with and without UC. A higher index represented greater separation between mean test results for patients with and without UC, and consequently greater accuracy when using a binary classification.

#### Data imputation method

A step-wise imputation technique was applied to impute all missing test values in the integrated dataset to obtain a new, imputed, comprehensive dataset (all variables, all sites). The dataset with the smallest number of missing values for a test was imputed first. This dataset was subsequently used to impute test variables in other datasets with the next smallest number of missing values, and so on until all missing values were imputed. If imputed values were not required in future imputations they were not used. Therefore, the maximum amount of known data was used to impute missing data, thus reducing imputation error.

Two different computational modelling approaches are used here to impute a missing value in a sample: personalized (individualized) modelling, and global modelling [7, 20]. To implement the personalized approach we used the *k*-nearest neighbour (*k*NN) method with different values for *k* (i.e. 3, 5, 10) (see Additional file 2).

To implement the global modelling approach we derived, from a subset of complete samples of selected input variables, a global function that was applied for the imputation of the missing test values. In this case the imputation was performed in terms of building a classification model of N inputs (the tests and variables with known values in a complete sub-set from the integrated data set) and one output – the test under imputation. Such a model was trained on input–output samples with known values and then recalled to calculate the unknown output values for the imputed test. Two global modelling methods were used in our experiments – multiple linear regression (MLR) and multi-layer perceptron (MLP) neural network (see Additional file 3).

#### Supervised versus unsupervised imputation

Data imputation was performed using two methods, depending on whether the known clinical truth (healthy or UC) was used (supervised) or not (unsupervised) during data imputation as an input variable. When unsupervised imputation was performed, missing values were imputed on the basis of outcomes reported for tests without taking into account the clinical truth. In contrast, when using a supervised imputation method, the clinical truth for each sample was considered as an input variable when the imputation function was derived and imputed values were calculated.

#### Assessment of diagnostic test accuracy – sensitivity and specificity

The true accuracy of each test in terms of how often the test outcome (negative or positive) matched the clinical truth (healthy or UC) was calculated as the probability of a positive test result (sensitivity) for a positive patient and negative test result (specificity) result for a negative patient.

$$ Sensitivity=\frac{TP}{TP+FN}\kern6em  Specificity=\frac{TN}{TN+FP} $$

wherein, TP and TN are the number of true positive and true negative results, respectively, and FN and FP are the number of false negative and false positive results, respectively. Univariate logistic regression was used to estimate the sensitivity and specificity as well as 95 % confidence intervals (CI) for each test using only the observed data.

#### Bayesian estimates of sensitivity and specificity

The MCMC methods [12] were used to estimate a probability model for the data. The measured data were allocated into either Tumor or Normal groups according to the cystoscopy result. For each group a multinomial model was used to assign a probability to each distinct set of each of the four binary test results, giving 16 probabilities for each group. We used uniform distributions on the interval (0,1) as priors for each of these probabilities. The transition probabilities for a Markov chain were constructed with a limiting distribution identical to joint conditional distribution of parameters given the observed data. Missing observations are on the same footing as parameters. Two thousand realizations from the Markov chain were simulated. Sensitivity is the marginal probability that a given test is positive in the Tumor group; similarly specificity is the marginal probability that a given test is negative for samples from the Normal group. Summary statistics were computed from these marginal distributions to obtain estimates and confidence intervals for sensitivities and specificities.

#### Assessment of the accuracy of the imputation techniques

The accuracy of each imputation method was first evaluated through cross-validation using the leave-one-out cross-validation technique (see [7]). For each imputation method and for each imputed diagnostic test, we used complete data samples to train and validate the method. The technique involves ‘taking out’ one complete sample for which the values of both input variables and the value of diagnostic test under imputation are known; then after applying the imputation method, the two values are compared and accuracy is calculated based on the ratio between the number of correctly imputed values and all imputation values. The lower the difference between the imputed data and the measured values, the closer the imputation matches known values. The leave-one-out cross validation technique is closer to the personalized modelling approach, when for every new individual data sample, we create a model to classify (predict) the outcome of this individual, using all available data samples of other individuals, and derive a personalized profile of the individual [21].

The SNR, sensitivity and specificity criteria were calculated again on the whole imputed data set. As another imputation evaluation procedure, the difference in sensitivity and specificity calculated in the imputed dataset and the integrated measured dataset for each diagnostic test and imputation method is calculated. If similar sensitivity and specificity values were achieved before and after imputation, the imputation method was considered to be consistent with the measured data, thus permitting further ranking of the tests and further study using the much larger integrated and imputed data that was subsequently available.

#### A novel integrated comparative analysis based on combined SNR, sensitivity and specificity evaluations before and after imputation

Each of the evaluation criteria – SNR, sensitivity and specificity, evaluates and ranks the UC diagnostic tests from a single point of view and using all of them together, rather than using only one of them, would be more appropriate when comparing the diagnostic tests in a holistic way. For example, SNR measures the discriminative power of a test, sensitivity measures probability of detecting UC, while specificity measures the probability of detecting healthy subjects. Different clinical laboratories may have different requirements according to their policy and goals. For an integrated comparison, our methodology includes a three-dimensional comparative analysis and ranking of the tests in the dimensions of the three criteria.

#### UC case study results presentation

All tests were compared and ranked according to the three criteria – SNR index, sensitivity and specificity, using data from the integrated dataset as measured and after supervised and unsupervised imputation of missing values.

## Results and discussion

The global integrated dataset comprised five contributing datasets and represented diagnostic test results for UC collected in different population studies. Patient samples lacking a diagnosis, or with only one test result available, were discarded; the remaining global integrated dataset comprised 939 samples (Table 1), including samples from 89 patients with UC and samples from 850 patients who did not have UC.

As indicated in Table 1, Datasets 1–4 did not have values for all UC diagnostic tests, but the clinical truth as determined by cystoscopy, was available for all samples. Only Dataset 5 was complete in terms of all tests being performed, but five individual values were missing. Altogether, values were missing for urine cytology in three patients, NMP22 in 162 patients, FISH in 622 patients and Cxbladder Detect in 193 patients. Age and gender data were available for all patients (see Table 2).

**Table 2:** Imputation process, in order of execution, for each of the datasets in the integrated dataset

Imputation step	Model inputs		Imputed variable output		
	Datasets	Variables	Dataset	Variable	Number of samples imputed
1	5	Age, gender, cytology, FISH, Cxbladder Detect	5	NMP22	2
2	5	Age, gender, NMP22, FISH, Cxbladder Detect	5	Cytology	3
3	1, 4, 5	Age, gender, cytology, NMP22	4	Cxbladder Detect	193
4	1, 2, 3, 4, 5	Age, gender, cytology, Cxbladder Detect	2	NMP22	80
			3	NMP22	80
5	3, 4, 5	Age, gender, cytology, Cxbladder Detect	3	FISH	68
6	2, 4, 5	Age, gender, cytology, Cxbladder Detect	2	FISH	80
7	1, 4, 5	Age, gender, cytology, Cxbladder Detect	1	FISH	474

Note: all imputations maintain at least a 70 % level of known data

The measured data in the integrated global dataset fell within the 95 % CI data published for sensitivity and specificity for cytology and NMP22 [21] and was very similar to the data in the single published study for Cxbladder Detect [18] (Table 3). The FISH measured dataset specificity was slightly higher than reported by Hajdinjak [22], and was slightly lower than Dimashkieh et al. [23] and Sullivan et al. [24]. In contrast, the FISH measured data in the integrated dataset sensitivity was lower than the range reported by Hajdinjak [22], but it was within the overall range of published values.

**Table 3:** Measured and published sensitivity and specificity for each test in the integrated dataset before imputation, mean and 95 % CIs

	Measured		Published	
	Sensitivity, % (95 % CI)	Specificity, % (95 % CI)	Sensitivity, % (95 % CI)	Specificity, % (95 % CI)
Cytology	45.5 (40.6–50.4)	96.3 (94.5–97.9)	56.1 (43.3–68.3) [18]	94.5 (91.9–96.5) [18]
NMP22	44.9 (37.4–52.3)	89.0 (86.5–91.5)	50.0 (37.4–62.6) [18]	88.0 (84.6–91.0) [18]
FISH	40.0 (22.7–52.3)	87.3 (83.7–91.6)	72 (69–75) [22]	83 (82–85) [22]
			61.9 [23]	89.7 [23]
			18 [24]	90 [24]
Cxbladder Detect	79.5 (71.1–87.8)	82.2 (79.2–85.0)	81.8 [18]	85.1 (fixed) [18]

First, a comparison of diagnostic tests using only measured data in the integrated global dataset was performed in a univariate analysis mode. The measured sensitivity and specificity of each test are presented in Table 3, along with their 95 % CIs and sensitivity and specificity values based on published data. Cxbladder Detect had a higher measured sensitivity of 79.5 % compared with cytology, FISH and NMP22, with sensitivity ranging from 40.0–45.5 %. However, urine cytology had a higher measured specificity at 96.3 % compared with specificities ranging from 82.2–89.0 % for Cxbladder Detect, FISH and NMP22. The 95 % CIs for the sensitivity of Cxbladder Detect (71.1–87.8 %) cover a higher range, and do not overlap those of the other three tests.

The results of the Bayesian analysis of sensitivity and specificity are presented in Table 4. Comparing these results with the measured sensitivities and specificities in Table 3, we see that the largest differences occur for FISH where sensitivity increases to 47.7 % (31.5–63.3 %) from 40.0 % (22.7–52.3 %) in Table 3, and for Cxbladder Detect where sensitivity decreases to 73.6 % (65.1–81.7 %) from 79.5 % (71.1–82.8 %) in Table 3. Although the Bayesian analysis imputation has raised the sensitivity of FISH and lowered the sensitivity of Cxbladder Detect, the Cxbladder Detect sensitivity remains significantly higher than FISH.

**Table 4:** Sensitivity and specificity for each test from the Bayesian estimate of conditional distribution of parameters and missing observations given observed data, mean and 95 % CIs

	Sensitivity, % (95 % CI)	Specificity, % (95 % CI)
Cytology	46.0 (36.3–55.8)	95.3 (93.7–96.6)
NMP22	45.9 (35.9–56.3)	88.0 (85.5–90.2)
FISH	47.7 (31.5–63.3)	87.7 (84.7–90.3)
Cxbladder Detect	73.6 (65.1–81.7)	81.7 (78.7–84.4)

**Fig. 1:**
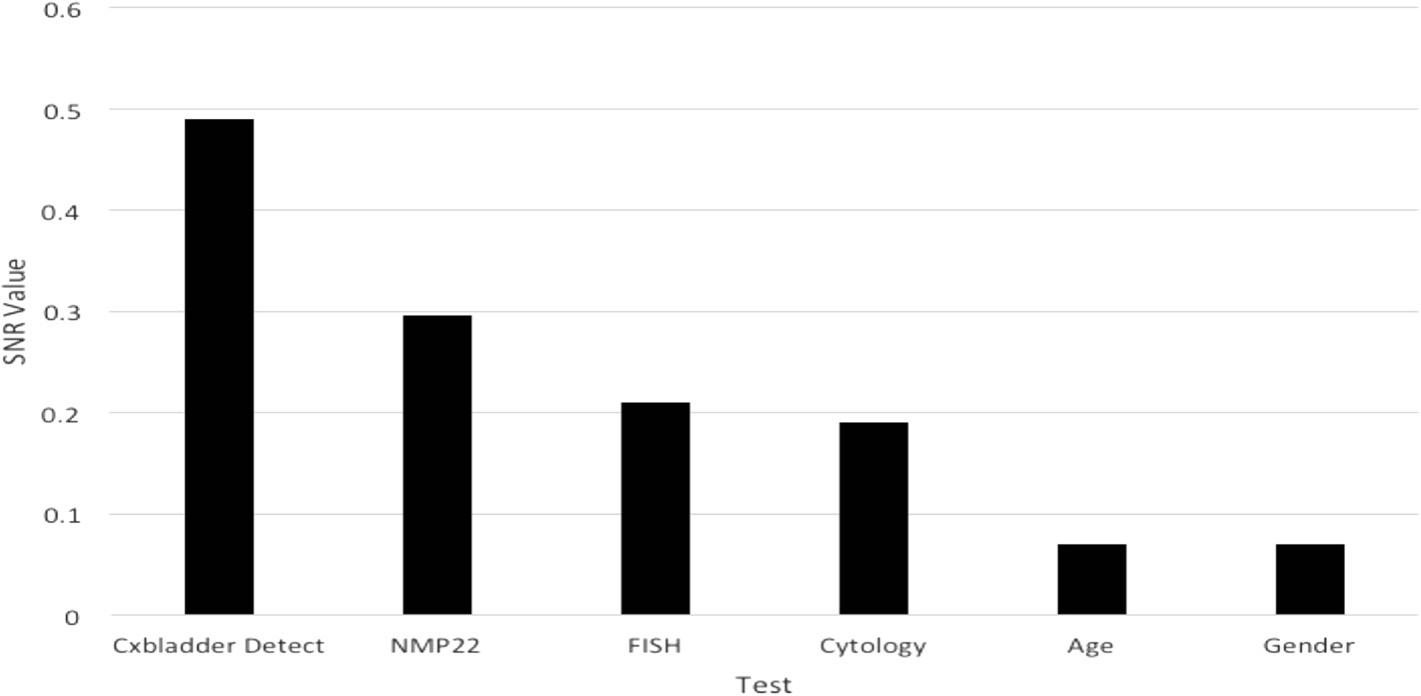
Ranking of tests in a univariate mode using SNR on the integrated dataset before imputation

When ranking the tests utilizing SNR using measured data alone in the integrated dataset, Cxbladder Detect offered the highest SNR of 0.48 compared with 0.21, 0.19 and 0.30 for FISH, urine cytology and NMP22, respectively (see Fig. 1). By comparison, age and gender offered a much lower SNR.

Supervised and unsupervised imputation was applied to the integrated dataset from Table 1 and the diagnostic tests were compared and ranked again. A step-wise summary of the 7-step process used to generate the global imputed dataset is described in Table 2. In Step 1, two values of the NMP22 test (output) were imputed in Dataset 5 using complete samples that included known values for age, gender, urine cytology, FISH and Cxbladder Detect as inputs. In the final, 7^th^ imputation step, 474 missing values of FISH were imputed in Dataset 1, using complete input samples for age, gender, urine cytology and Cxbladder Detect from Datasets 1, 4 and 5.

After both supervised and unsupervised imputation was performed using the three variations of the *k*NN method (*k* = 3, 5 and 10), and two global modelling methods (MLR and MLP), the accuracy of the tests was again calculated in terms of sensitivity and specificity across the whole imputed data set (Table 5).

**Table 5:** Sensitivity and specificity of tests measured on the integrated, imputed dataset using different imputation methods

	Supervised imputation		Unsupervised imputation	
	Sensitivity, %	Specificity, %	Sensitivity, %	Specificity, %
**3NN**				
Cytology	44.94	96.35	44.94	96.35
NMP22	41.57	88.82	41.57	88.94
FISH	32.58	85.24	38.20	85.95
Cxbladder Detect	80.90	82.12	77.53	79.76
**5NN**				
Cytology	44.94	96.35	44.94	96.35
NMP22	43.82	90.26	39.33	90.24
FISH	29.21	91.90	31.46	91.55
Cxbladder Detect	80.90	83.80	77.53	80.47
**10NN**				
Cytology	44.94	96.49	44.94	96.35
NMP22	43.82	90.59	39.33	90.35
FISH	29.21	91.07	23.60	90.00
Cxbladder Detect	80.90	85.53	78.65	82.59
**MLR**				
Cytology	44.94	96.35	44.94	96.56
NMP22	42.70	90.82	39.33	90.82
FISH	47.19	93.69	47.19	93.33
Cxbladder Detect	80.90	77.29	77.53	84.71
**MLP**				
Cytology	44.94	96.35	44.94	96.35
NMP22	39.33	90.82	39.33	90.82
FISH	49.44	93.81	47.19	93.33
Cxbladder Detect	78.65	85.18	77.53	84.71

Each imputation method was cross-validated using the leave-one-out method. Specifically, the accuracy of the imputed values were compared with the measured values from the dataset before it was used to impute the unknown values for the same test. The cross-validation results along with the difference between the sensitivity and specificity values calculated before and after imputation, are given in Table 6. Cross-validation accuracy was >81 % for all of the imputed tests across all imputation methods. The upper limits of the difference between sensitivity and specificity of the integrated data before and after imputation was <6 % for Cxbladder Detect, cytology and NMP22 and <16 % for FISH for all imputation methods (see Table 6). This demonstrated that the imputed data was consistent with the measured data across all tests and the missing values derived through imputation were valid. Tables 5 and 6 can be used to derive some conclusions about the imputation methods in relation to the type of the imputed tests; for example, from Table 6 it can be said that the most appropriate imputation method with a combined objective function of both high cross-validation accuracy and low average difference between the sensitivity/specificity evaluated before and after the imputation was the 3NN model in the supervised imputation mode. From Table 5 it can be concluded that both supervised and unsupervised imputation using MLP and MLR bring the FISH sensitivity to the highest value of 49.44 % and 47.19 %, respectively. It should be noted that the ratio of positive to negative patients is lowest in Dataset 4, and overall confirmed positive patients with collected FISH data from Dataset 4 and Dataset 5 total 15. Consequently, relatively large numbers of imputed positive data points and the cumulative nature of the imputation methodology may have relatively large effects on the performance characteristics of the FISH test.

**Table 6:** Cross-validation of methods and difference between sensitivity and specificity obtained before and after imputation

	Supervised imputation				Unsupervised imputation			
Imputation method	Leave-one-out cross-validation accuracy of the imputation, %	Sensitivity difference before and after imputation, %	Specificity difference before and after imputation, %	Average absolute difference before and after imputation, %	Leave-one-out cross-validation accuracy of the imputation, %	Sensitivity difference before and after imputation, %	Specificity difference before and after imputation,%	Average absolute difference before and after imputation, %
**3NN**								
Cytology	96.70	2.68	0.02	1.35	96.70	2.68	0.02	1.35
NMP22	83.44	3.30	0.41	1.86	83.19	3.30	0.29	1.80
FISH	83.17	7.42.	1.70	4.56	85.49	1.80	0.99	1.40
Cxbladder Detect	81.44	−1.38	0.67	1.03	80.44	1.99	3.03	2.51
Mean for method	86.19	3.01	0.70	2.20	86.46	2.44	1.08	1.76
**5NN**								
Cytology	96.69	2.68	0.02	1.35	96.69	2.68	0.02	1.35
NMP22	84.20	1.05	−1.03	1.04	84.07	5.54	−1.01	3.28
FISH	84.54	10.79	−4.96	7.88	85.80	8.54	−4.61	6.58
Cxbladder Detect	81.94	−1.38	−1.01	1.20	81.10	1.99	2.32	2.16
Mean for method	86.84	3.29	−1.75	2.87	86.92	4.69	−0.82	3.34
**10NN**								
Cytology	96.69	2.68	0.02	1.35	96.69	2.68	0.02	1.35
NMP22	86.09	1.05	−1.36	1.21	85.84	5.54	−1.12	3.33
FISH	86.12	10.79	−4.13	7.46	85.80	16.40	−3.06	9.73
Cxbladder Detect	82.44	−1.38	−2.74	2.06	81.77	0.87	0.20	0.53
Mean for method	87.84	3.29	−2.05	3.02	87.53	6.37	−0.99	3.74
**MLR**								
Cytology	96.69	2.68	0.02	1.35	96.69	2.68	0.02	1.35
NMP22	85.59	2.17	−1.59	1.88	85.21	5.54	−1.59	3.56
FISH	89.58	−7.19	−6.75	6.97	89.58	−7.19	−6.39	6.79
Cxbladder Detect	84.11	−1.38	5.50	3.44	84.20	1.99	−1.92	1.95
Mean for method	88.99	−0.93	−0.70	3.41	88.92	0.75	−2.47	3.42
**MLP**								
Cytology	95.87	1.55	0.02	0.79	95.87	2.68	0.02	1.35
NMP22	86.35	5.54	−1.59	3.56	83.19	5.54	−1.59	3.56
FISH	89.25	−9.44	−6.87	8.16	88.60	−7.19	−6.39	6.79
Cxbladder Detect	82.78	0.87	−2.39	1.63	82.47	1.99	−1.92	1.95
Mean for method	88.56	−0.37	−2.71	3.53	87.53	−2.47	−2.47	3.42

Difference = measured – imputed

In many cases of imputation the difference between supervised and unsupervised imputation in terms of sensitivity and specificity calculated on the whole integrated and imputed datasets is small (see Tables 5 and 6). This indicates that the input variables used for imputation carry, in their integration and interaction, sufficient information about the clinical truth and adding the clinical truth as an additional input variable does not materially affect the imputation process.

**Fig. 2:**
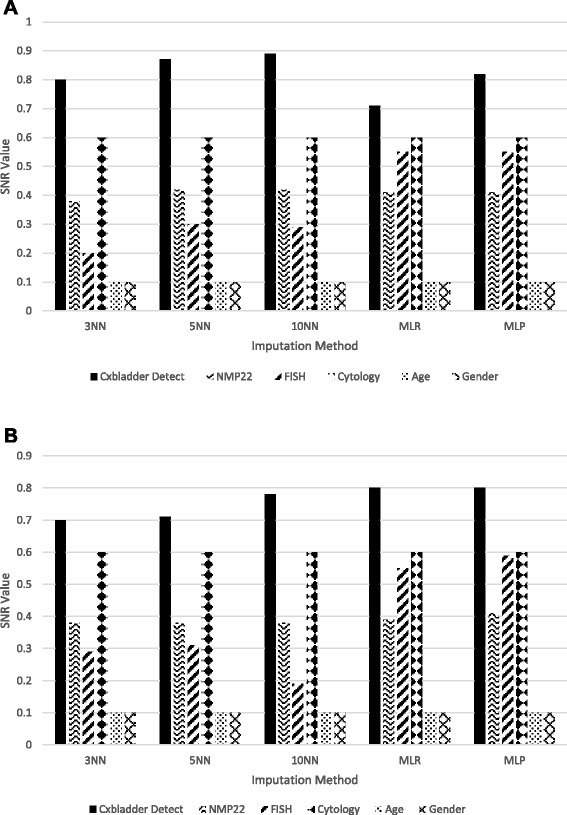
Rankings of tests for the integrated dataset after **a** supervised and **b** unsupervised imputation

After the validity of the imputed data was confirmed, the tests were re-ranked using the global imputed dataset (supervised and unsupervised) using the same SNR method as for the measured data (Fig. 2). Cxbladder Detect consistently outperformed all other tests across all methods of data imputation, followed by urine cytology, FISH and NMP22. The relative performance of FISH and NMP22 was lower than Cxbladder Detect and cytology, but their rankings varied across data imputation methodologies. In contrast, age and gender demonstrated little usefulness in separating patients with or without UC. Notably, SNR rankings derived from the measured data were comparable with the rankings observed following all forms of data imputation and the global imputed dataset exhibited higher overall SNR values. These rankings were consistent with other methods that indicated that Cxbladder Detect was the highest ranked UC diagnostic test.

The same relative global ranking of the tests is achieved when ranking UC diagnostic tests using the non-imputed integrated and imputed integrated datasets: Cxbladder Detect consistently outperformed urine cytology, FISH and NMP22 in terms of overall discriminative power to separate UC from healthy samples. This is in agreement with Cxbladder Detect’s higher sensitivity to detect UC in both measured and imputed datasets.

**Fig. 3:**
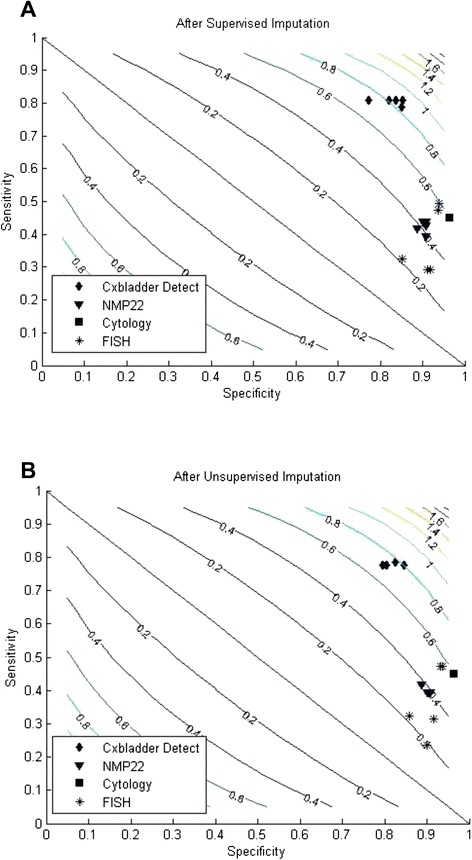
Comparisons after **a** supervised and **b** unsupervised imputation in two-dimensional contour plots of sensitivity and specificity

Two dimensional contour plots of sensitivity and specificity using the whole imputed dataset, either supervised or unsupervised, are shown in Fig. 3a, b, respectively. Both plots show clear data clusters, with Cxbladder Detect separated from the other tests, that cluster together. The separation is largely due to the higher sensitivity of the Cxbladder test compared with the other tests.

As this comparison is between populations with a known diagnosis, supervised imputation was a viable option and was investigated in an attempt to factor out the imbalance between data from patients with and without UC without the need to apply over- or under-sampling techniques, thus preserving the nature of the original data. When resampling techniques were initially investigated to determine their viability in this study it was found that the sensitivity values, in particular, were noticeably reduced, as the number of clinically true UC samples was comparatively small. Specificity was also reduced, but to a lesser extent. For example, using a resampling rate of 300 % and the 3NN imputation method, the results for FISH in particular, which was the most susceptible to the influence of resampling on sensitivity, was only 8.4 % compared with the non-resampled imputation results of 38.2 % and 32.6 % for unsupervised and supervised imputation, respectively. From these results it was determined that resampling resulted in no positive effect on imputation and the technique discarded. Supervised imputation is a liberal approach, where the tests with the most missing values, or the worst ranking in the measured integrated dataset, are likely to demonstrate the greatest relative improvement in accuracy after imputation, offering higher accuracy than unsupervised imputation. However, supervised imputation may not reflect the true accuracy levels of each test because unsupervised imputation allows the new values to be calculated independently of the clinical outcome. In contrast, unsupervised imputation constitutes a conservative approach that does not use the whole available information and reflects the normal error levels for each test better than supervised imputation, whilst also acknowledging that the unsupervised version is more volatile with respect to the true clinical outcome, but not the variability of the tests.

The number *k* of nearest neighbour samples referenced for imputation may affect the accuracy of the imputed values when *k*NN methods are used (see Tables 5 and 6). The number *k* may be optimized for each of the tests. The more the integrated data is balanced, in terms of having a similar number of samples from healthy subjects and subjects with UC, the lower the influence of *k*. In terms of imbalanced data, using the clinical truth value as an input variable for imputation would be appropriate.

The concept of holistic is fundamental to the methodology and interpretation presented in this study. Holistic assumes that all contributing datasets are able to be analyzed and interpreted as one population both, in this case, for imputation of missing values and interpretation of the diagnostic data. However, in the this study the contributing datasets were different in terms of patient origin (Australasia and USA), primary detection or secondary monitoring of UC and the degree of imputation of the various tests – FISH was most imputed and affected, and in contrast, Cxbladder Detect and cytology were least imputed and least affected by imputation. Hence these assumptions require some justification and any bias considered before imputation and interpretation can take place. In this combined dataset, we assume that all data were from either patients with UC or patients presenting with hematuria and origin is of lower importance than disease status, so no bias is likely to arise. Primary and secondary (recurrence) of UC share the same stage and grade categories [25] and so we assume for this study that tests are equally diagnostic at each part of cancer progression and no bias exists. In contrast, imputation bias affects each diagnostic test differently: FISH being most imputed and affected. However, the cross-validation accuracy analysis (Table 6) reveals that imputation for FISH was as accurate as NMP22 and more accurate than Cxbladder Detect. Moreover, imputation using MLR and MLP actually improved sensitivity and specificity estimated using the FISH data. This means that the apparent imputation bias was unlikely to have adversely affected the overall appraisal of the FISH diagnostic data, particularly its relative ranking. Nevertheless these assumptions are critical to the overall ranking comparisons after missing value imputation. If the assumptions are accepted and missing values can be imputed, then datasets can be used to rank diagnostic tests and hence permit comparisons of relative diagnostic merit as calculated in this case by SNR analysis.

## Conclusions

The proposed methodology, applied here on UC diagnostic tests comparative analysis and ranking, showed a significant advantage of the Cxbladder Detect versus other UC diagnostic tests. It can be applied in the future for a comprehensive comparative analysis and global ranking of other cancer diagnostic and prognostic tests and multiple cancer diagnostics [26].

## Additional files

Additional file 1:
**Signal-to-noise ratio (SNR) univariate ranking method.**
Additional file 2:
***k*** **nearest neighbour** **(** ***k*** **NN) classification methods for personalised modelling.**
Additional file 3:
**Global function classification methods: MLR and MLP.**

## Abbreviations

CI: Confidence interval
ELISA: Enzyme-linked immunosorbent assay
FISH: Fluorescence *in situ* hybridization
MCMC: Markov chain MonteCarlo
MLP: Multi-layer perceptron
MLR: Multiple linear regression
NN: Nearest-neighbour
SNR: Signal-to-noise ratio
UC: Urothelial carcinoma
