# Supplementary material for: A holistic comparative analysis of diagnostic tests for urothelial carcinoma: a study of Cxbladder Detect, UroVysion® FISH, NMP22® and cytology based on imputation of multiple datasets
Source: BMC Med Res Methodol. 2015 May 12;15:45. doi: 10.1186/s12874-015-0036-8 (PMC4494166; doi:10.1186/s12874-015-0036-8)
Supplement: Additional file 1: — Signal-to-noise ratio (SNR) univariate ranking method. [file 12874_2015_36_MOESM1_ESM.docx]

**Additional file 1. Signal-to-noise ratio (SNR) univariate ranking method**

In a two class problem for variable *x* the SNR ranking coefficient is calculated as the absolute difference of the two class means, divided by the sum of the standard deviations:

$$SNR\left( x \right)= \frac{\left| \mu_{1}-\mu_{2} \right|}{\sigma_{1}+ \sigma_{2}}$$
