# Supplementary material for: A holistic comparative analysis of diagnostic tests for urothelial carcinoma: a study of Cxbladder Detect, UroVysion® FISH, NMP22® and cytology based on imputation of multiple datasets
Source: BMC Med Res Methodol. 2015 May 12;15:45. doi: 10.1186/s12874-015-0036-8 (PMC4494166; doi:10.1186/s12874-015-0036-8)
Supplement: Additional file 2: — k nearest neighbour ( k NN) classification methods for personalised modelling. [file 12874_2015_36_MOESM2_ESM.docx]

**Additional file 2.** ***k* nearest neighbour (*k*NN) classification methods for personalised modelling**

A new sample (n-dimensional data point) was placed (compared) using all its attributes against its closest *k* nearest neighbours that had their class labels known, to determine, by majority, to which class the new data point was most likely to belong. The number of neighbours *k* checked can be varied, and is often an odd number so that a true majority will always arise. The similarity can be measured in a number of ways, the simplest of these is the Euclidian distance shown below on two n-dimensional vectors x and y, where the larger the distance – the lower the similarity [1]:

$$\left\| x-y \right\|=\left[ \frac{1}{n}\sum_{j=1}^{n} \left| x_{j}-y_{j} \right|^{2} \right]^{\frac{1}{2}}$$

**Reference**

1. Kasabov N. Global, local and personalised modelling and profile discovery in bioinformatics: an integrated approach. Pattern Recognit Lett. 2007;28:673-85.
