# Supplementary material for: A holistic comparative analysis of diagnostic tests for urothelial carcinoma: a study of Cxbladder Detect, UroVysion® FISH, NMP22® and cytology based on imputation of multiple datasets
Source: BMC Med Res Methodol. 2015 May 12;15:45. doi: 10.1186/s12874-015-0036-8 (PMC4494166; doi:10.1186/s12874-015-0036-8)
Supplement: Additional file 3: — Global function classification methods: MLR and MLP. [file 12874_2015_36_MOESM3_ESM.docx]

**Additional file 3.** **Global function classification methods: MLR and MLP**

A global function classification method derives a single function from training input-output data vectors that can be used to calculate unknown values for the output, when input values are provided.

**Multiple linear regression (MLR)** calculates a linear regression expression for each output class from training data. The input variables are defined in Table 3. For a new sample the linear expression for each class is calculated, and the one found to have the highest value is selected as the output class. This method is sometimes also known as multiresponse linear regression as it can deal with multiple class values.

**Multilayer perceptron (MLP)** is a classical neural network method that can be used to build a classification model from data. In a 3-layer MLP the model consists of inputs, ‘hidden nodes’ and outputs. The error back-propagation algorithm is used to train the model on input-output data through changing the connections between the neurons (nodes) in the model so that the output error between the given output values and the derived from the trained model is minimized [1]. In the imputation of the UC diagnostic tests from Table 5 the following parameters of a MLP are used: number of input nodes is 5 or 4 according to the number of input variables used (see Table 2); number of hidden nodes is 9; number of training iterations is 200; learning rate is 0.1.

**Reference**

1. Kasabov N. Evolving connectionist systems: The knowledge engineering approach. Second edition. London: Springer-Verlag; 2007.
